# The association between SGLT2 inhibitor use and diabetic peripheral neuropathy in type 2 diabetes: a cross-sectional study

**DOI:** 10.3389/fendo.2026.1860467

**Published:** 2026-07-24

**Authors:** Yuwei Xing, Yishan Yin, Qianqian Zhao

**Affiliations:** 1 Department of Endocrinology, The Second Hospital of Shijiazhuang, Shijiazhuang, China; 2 Department of Orthopedics, The Armed Police Forces Hospital of Shandong, Jinan, China

**Keywords:** cross-sectional study, diabetic peripheral neuropathy, propensity score matching, SGLT2 inhibitor, type 2 diabetes

## Abstract

**Introduction:**

This study assessed the association between sodium-glucose cotransporter 2 (SGLT2) inhibitor use and the prevalence of diabetic peripheral neuropathy (DPN) in patients with type 2 diabetes mellitus (T2DM).

**Methods:**

We classified 2, 456 individuals with T2DM according to SGLT2 inhibitor treatment status. Propensity score matching (PSM) was implemented as the primary adjustment technique to achieve balance between groups on baseline demographics, laboratory values, comorbidities, and concurrent glucose-lowering agents. Additional propensity score weighting approaches were employed to examine the robustness of our findings, along with subgroup analyses and E-value calculations.

**Results:**

Among all participants, 399 (16.2%) had received SGLT2 inhibitors. Following PSM, each study arm comprised 398 patients. In the matched cohort, SGLT2 inhibitor use was associated with significantly increased odds of DPN (odds ratio 1.55; 95% confidence interval 1.17–2.05; p = 0.002). This association remained consistent across different propensity score methods, with odds ratios ranging from 1.55 to 1.7 (all p < 0.01). Subgroup analyses confirmed statistical significance, and E-value assessment supported the robustness of the observed effect.

**Conclusion:**

SGLT2 inhibitor use was associated with a higher prevalence of DPN. Further prospective studies are warranted to establish causality.

## Graphical abstract

**Figure:**
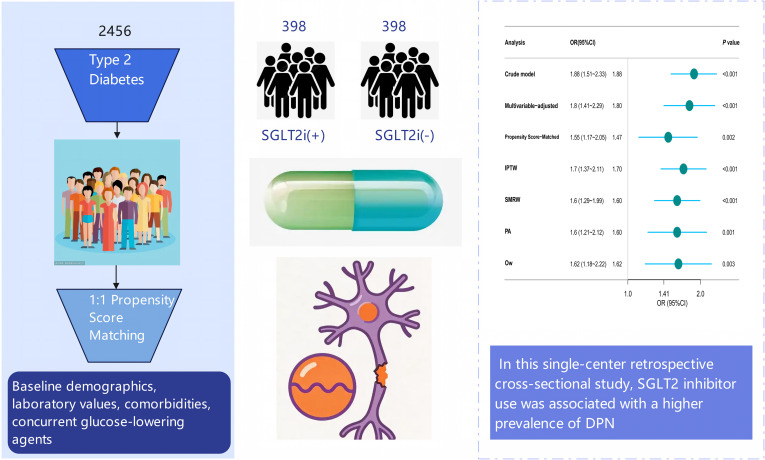

## 1 Introduction

T2DM (type 2 diabetes mellitus) (1) has emerged-health challenges of the 21st century. As a chronic, progressive condition, its microvascular complications are major causes of reduced life quality, long-term disability and premature death. The most frequent and disabling of these complications is diabetic peripheral neuropathy (DPN) (2), which produces pain, numbness and sensory deficits and is the principal antecedent of diabetic foot ulcers and lower-extremity amputation, thereby placing a heavy burden on patients and healthcare systems (3). Global estimates indicate that roughly half of people with diabetes will develop DPN during their lifetime, and given the rising prevalence of diabetes, the population burden of DPN is projected to grow further in coming decades (2). The pathogenesis of DPN is multifactorial and not yet fully clarified: hyperglycemia-driven processes such as increased polyol pathway flux, accumulation of advanced glycation end-products (AGEs), oxidative stress, mitochondrial dysfunction and microvascular injury synergize to injure peripheral neurons and Schwann cells (4, 5). Although elevated HbA1c and longer diabetes duration are the best-established risk factors, a substantial amount of DPN risk remains even among patients with good glycemic control, suggesting additional glucose-independent mechanisms — for example metabolic memory, disturbances in lipid metabolism, and possible drug-specific effects (2, 5, 6).

Contemporary guidelines from the American Diabetes Association (ADA 2026) position sodium–glucose cotransporter-2 inhibitors (SGLT2i) as important adjunctive agents in the management of type 2 diabetes, particularly for patients with or at high risk for cardiorenal disease (7). Specifically, the ADA 2026 guidance recommends that SGLT2i be considered for individuals with T2D and established atherosclerotic cardiovascular disease, heart failure, or chronic kidney disease (independent of baseline glycaemic control), and more broadly for patients at high risk of these complications (7–9). Preclinical data and secondary analyses of outcome trials indicate that, aside from lowering glucose, reducing weight and decreasing blood pressure, these agents may exert neuroprotective effects through anti−inflammatory actions, mitigation of oxidative stress, and enhancement of mitochondrial function (10, 11). In rodent models, SGLT2i have been reported to lessen apoptosis in dorsal root ganglia and to improve nerve conduction velocities (12).

Although laboratory and mechanistic data are promising, epidemiological research on whether SGLT2 inhibitors influence DPN risk remains sparse and contradictory. Some have postulated neuroprotection owing to the drugs’ diverse benefits (10), while others raise concerns that rapid correction of hyperglycemia can provoke treatment−related neuropathy and that weight loss may aggravate neuropathic states (13). Only a handful of observational studies have tackled this issue, frequently limited by small cohorts, incomplete confounder adjustment and inconsistent results. Notably, large baseline differences in demographics, comorbidities and concurrent medications between SGLT2i users and comparators generate selection and residual confounding that standard multivariable models may not adequately control.

Although large randomized cardiovascular and renal outcome trials have established that SGLT2i reduce major adverse cardiovascular and renal outcomes, these trials did not systematically evaluate diabetic peripheral neuropathy (DPN) as a prespecified or adjudicated outcome; moreover, trial populations, follow-up intervals, and endpoint definitions limit inferences about neuropathic risk in routine clinical practice (14).

Randomized controlled trials (RCTs) remain the gold standard for causal inference regarding prespecified endpoints; however, peripheral neuropathy was not a primary endpoint in the major SGLT2i trials, and RCT eligibility criteria and follow−up duration may limit detection of neuropathic outcomes. Well−conducted observational analyses can therefore complement trial evidence by assessing neuropathy outcomes in broader, more representative populations and over longer time horizons, provided that advanced methods (new−user design, active comparators, propensity score techniques and multiple sensitivity analyses) are used to mitigate confounding.

Therefore, whether a robust and consistent association exists between SGLT2i use and the prevalence of DPN represents a critical knowledge gap that warrants rigorous investigation. Addressing this gap is of paramount importance for informing clinical decision-making and optimizing strategies for neuropathy prevention and management in patients with T2DM.

The objective of this study was to evaluate the association between SGLT2 inhibitor use and the prevalence of DPN in a large, contemporary sample of patients with type 2 diabetes, employing a cross-sectional design with robust confounding control through propensity score methods. We further assessed the robustness of our findings through subgroup analyses and E-value calculations.

## 2 Materials and methods

### 2.1 Study design and participants

This study was a single-center cross-sectional study conducted at the Department of Endocrinology of the Second hospital of Shijiazhuang. Eligible participants from January 2024 to December 2025 were initially identified via electronic medical records and standardized ICD-10 diagnostic coding, with target cases defined as patients with a confirmed diagnosis of type 2 diabetes mellitus; among these patients, diabetic peripheral neuropathy was ascertained using ICD-10 code E11.4, supplemented by attending physician diagnoses, objective confirmatory testing, and compatible neuropathic symptoms documented in the clinical notes, as detailed in the Methods. This study strictly adhered to the guidelines of the Declaration of Helsinki and was approved by the Ethics Review Committee of the Second Hospital of Shijiazhuang City (Ethics Approval number:SEY202011117). Because the study was retrospective, and all participant data were fully anonymized, the ethics committee waived the requirement for informed consent.

### 2.2 Diagnostic criteria for diabetic peripheral neuropathy

Diabetic peripheral neuropathy was assessed in accordance with the Guideline for the Prevention and Treatment of Diabetes Mellitus in China (15)(2024 Edition) issued by the Chinese Diabetes Society. Distal symmetric polyneuropathy (DSPN) was screened using the five-item clinical examination protocol recommended by the guideline: (i) ankle reflex testing; (ii) 128-Hz tuning fork vibration perception; (iii) 10-g monofilament pressure sensation; (iv) pinprick pain perception; and (v) temperature discrimination. The presence of two or more abnormal findings among these five tests defined clinical DSPN; fewer than two abnormal findings classified participants as without DSPN. This binary classification follows the guideline’s standard screening algorithm for clinical and epidemiological settings. Nerve conduction studies were performed only in atypical presentations or when alternative etiologies (e.g., vitamin B12 deficiency, cervical/lumbar radiculopathy, chronic inflammatory demyelinating polyneuropathy, uremic or drug-induced neurotoxicity) required exclusion. Small-fiber neuropathy was evaluated through quantitative sensory testing (thermal thresholds) in participants with negative large-fiber tests but typical neuropathic symptoms (16, 17).

Patients were excluded if an alternative explanation for neuropathy existed, including cervical or lumbar spine disease, vitamin B12 deficiency, chronic inflammatory demyelinating polyneuropathy, hereditary neuropathy, advanced peripheral vascular disease, end-stage renal disease requiring maintenance dialysis, use of neurotoxic medications, or incomplete QST data.

### 2.3 Standardized data collection and neurological assessment

All clinical neurological assessments, including the 10−g monofilament test, 128−Hz vibration perception test, ankle jerk reflex examination, and standardized DPN-related symptom questionnaires, were performed and documented by well-trained research personnel who had completed a unified training program and strictly adhered to a standardized operating procedure (SOP) to ensure inter-rater reliability and consistency across all assessments.

### 2.4 Definition of key exposure variables

SGLT2 inhibitor use was categorized as a binary variable (yes/no) based on medical prescription records and patient medication logs. During the study period, only dapagliflozin and empagliflozin were available on the hospital formulary and prescribed to patients at our hospital; no other SGLT2 inhibitors (e.g., canagliflozin, ertugliflozin) were dispensed.

### 2.5 Inclusion and exclusion criteria

#### 2.5.1 Inclusion criteria

Age ≥18 years at the time of enrollment; Individuals were classified as having diabetes if they fulfilled one or more of the following items: characteristic symptoms of diabetes accompanied by a random venous plasma glucose level ≥ 11.1 mmol/L; a fasting state plasma glucose concentration ≥ 7.0 mmol/L; or a plasma glucose value ≥ 11.1 mmol/L measured 2 hours after a 75-g oral glucose tolerance test (OGTT), Confirmatory testing on a separate day is mandatory for asymptomatic individuals. Confirmed diagnosis of type 2 diabetes mellitus based on the criteria of the American Diabetes Association (ADA) or World Health Organization (WHO) (18).

#### 2.5.2 Exclusion criteria

Exclusion criteria were type 1 diabetes, gestational diabetes, other specific types of diabetes, acute diabetic complications. Confirmed diagnosis of non-diabetic peripheral neuropathy, including hereditary motor and sensory neuropathy, severe alcoholic peripheral neuropathy, HIV-associated neuropathy, toxic neuropathy, or other secondary neuropathy unrelated to diabetes; Acute onset peripheral neuropathy within 3 months prior to enrollment; Uncorrected severe vitamin B12 deficiency, a known reversible cause of peripheral neuropathy; End-stage renal disease requiring maintenance dialysis; Inability to complete standardized neurological assessments due to severe lower extremity lesions (e.g., amputation, severe ulceration, deformity) or significant cognitive impairment that precluded reliable symptom reporting and physical examination cooperation.

### 2.6 Covariates

The system collects and organizes the general clinical data of patients, covering indicators such as sex, age, smoking history, height, weight, duration of diabetes, diabetic retinopathy, concurrent hypertension and concurrent cerebral infarction. The participants’ body weight and height were recorded while they wore light indoor clothing and were barefoot. A calibrated electronic column scale (Omron, China) was used to obtain weight measurements that were accurate to 0.1 kg. Height was determined to the nearest 0.1 cm by employing a wall-mounted stadiometer. Subsequently, the body mass index (BMI) was derived from the formula: weight (in kilograms) divided by height (in meters) squared (kg/m²).

Diabetes duration was defined as the time interval from the first documented diagnosis of diabetes in the electronic medical record system to the date of study enrollment. For patients without a recorded diagnosis date, diabetes duration was supplemented by patient self-reported history documented in clinical notes.

Ischemic heart disease (IHD) was defined as the presence of coronary atherosclerotic disease documented before the index date; Diabetic kidney disease (DKD) was defined as a clinical diagnosis documented in the medical record.

Venous blood specimens were obtained from each participant the morning after they had fasted for a minimum period of 8 h overnight. The analyzed biochemical parameters included glycated hemoglobin (HbA1c), fasting blood glucose (FBG), estimated glomerular filtration rate (eGFR, calculated via the CKD-EPI formula based on enzymatically measured serum creatinine), triglycerides, total cholesterol, high-density lipoprotein (HDL) cholesterol, and low-density lipoprotein (LDL) cholesterol.

Concurrent medications included biguanides, sulfonylureas, α-glucosidase inhibitors, thiazolidinediones, GLP-1 analogues, DPP-4 inhibitors and insulin, categorized as use or non-use, with data verified through medical prescription records, pharmacy dispensing data, and patient medication logs. All covariate data were collected by designated personnel and subjected to double-entry verification to minimize data entry errors.

### 2.7 Statistics

Baseline continuous variables judged to be approximately normally distributed are shown as mean ± SD, while those with skewed distributions are summarized by median and IQR; categorical variables are presented as counts (percentages). Between−group testing employed t−tests for parametric continuous variables, Mann–Whitney U tests for nonparametric continuous variables, and χ2 or Fisher’s exact tests for categorical variables. A two−sided P−value <0.05 indicated statistical significance.

**Figure 1:**
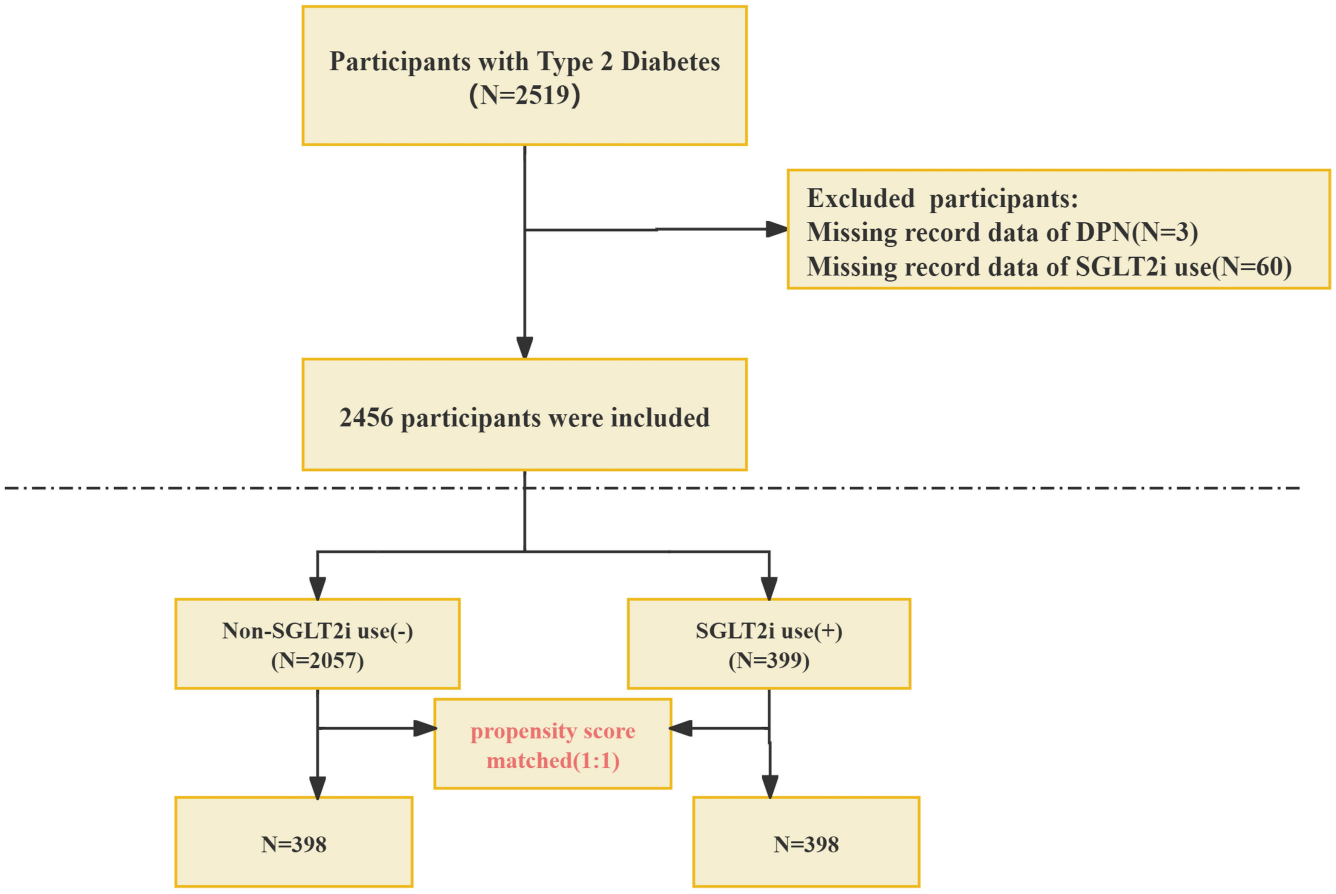
Flowchart of participant selection and study design. Flow diagram illustrating the participant selection process. A total of 2, 519 patients with type 2 diabetes were initially screened. After excluding participants with missing data on diabetic peripheral neuropathy (DPN, n=3) or SGLT2 inhibitor use (n=60), 2, 456 participants were included in the final analysis. Participants were categorized into two groups based on SGLT2 inhibitor use: non-SGLT2i group (n=2, 057) and SGLT2i group (n=399). To minimize confounding bias, 1:1 propensity score matching was performed, resulting in 398 participants in each group for subsequent analyses. DPN, diabetic peripheral neuropathy; SGLT2i, sodium-glucose cotransporter 2 inhibitor.

We initially screened 2, 519 patients with type 2 diabetes; after excluding 3 with missing DPN data and 60 lacking information on SGLT2 inhibitor exposure, 2, 456 subjects remained for analysis, of whom 399 (16.2%) were SGLT2 inhibitor users and 2, 057 (83.8%) were non−users (**Figure 1**).

Cases with absent exposure or outcome data were excluded. Missing data were handled using multiple imputation by chained equations (MICE) via the mice package in R (19), employing fully conditional specification with models tailored to variable type (predictive mean matching for continuous, logistic regression for binary, polytomous regression for unordered categorical). Five imputed datasets were generated to ensure adequate between-imputation variability. Imputation quality and convergence were assessed through diagnostic plots. All logistic regression analyses were performed separately within each imputed dataset and pooled using Rubin’s rules. For propensity score matching, the primary analysis employed a dataset created by averaging across the imputed propensity scores—a Rubin-compatible strategy for synthesizing multiply imputed estimates, not an arbitrary selection of one imputation. For the sensitivity analysis of propensity score matching (PSM), 1:1 nearest-neighbor matching without replacement was performed independently within each of the five imputed datasets, and the resulting estimates were pooled across the five datasets using Rubin’s rules. The consistency of results across all five imputed datasets supports the robustness of the primary finding.

To ensure the stability of the model, before conducting the final analysis, we conducted a multicollinearity diagnosis for all the covariates included in the model and calculated the variance inflation factor (VIF). The results showed that except for the VIF value of total cholesterol (CHOL), which was 7.96 and reached the critical level of multicollinearity, the VIF values of all other covariates were between 1.01 and 4 (**Supplementary Figure 1**). To avoid potential bias in the estimates caused by multicollinearity, we decided to remove CHOL from the final propensity score model.

To mitigate confounding by indication, we derived propensity scores for SGLT2 inhibitor exposure using a logistic regression that incorporated all covariates listed in **Table 1** (demographics, laboratory indices, comorbid conditions and concurrent glucose−lowering therapies). Subjects were then matched 1:1 on the propensity score, producing 398 matched pairs; balance was evaluated by standardized mean differences. Sensitivity analyses employed alternative propensity−score methods—continuous propensity−score adjustment, IPTW, SMRW and OW. The primary endpoint was prevalent DPN, and estimates are reported as odds ratios (ORs) with 95% confidence intervals (CIs).

**Table 1:** Baseline characteristics of the study population stratified by SGLT2 inhibitor use.

Variable	Total (n = 2456)	Non-SGLT2i group (n = 2057)	SGLT2i group (n = 399)	P value
Demographic characteristics				
Sex, n (%)				< 0.001
Male	1335 (54.4)	1066 (51.8)	269 (67.4)	
Female	1121 (45.6)	991 (48.2)	130 (32.6)	
Age (years), Mean ± SD	62.8 ± 11.5	63.1 ± 11.6	61.3 ± 10.9	0.004
BMI (kg/m²), Mean ± SD	26.3 ± 5.9	26.3 ± 6.2	26.1 ± 3.6	0.633
Diabetes duration (years), Median (IQR)	2.0 (1.0, 6.0)	2.0 (1.0, 6.0)	2.0 (1.0, 6.5)	0.549
FHD, n (%)				0.231
No	2027 (82.5)	1706 (82.9)	321 (80.5)	
Yes	429 (17.5)	351 (17.1)	78 (19.5)	
Smoking history, n (%)				0.131
No	2063 (85.8)	1738 (86.3)	325 (83.3)	
Yes	342 (14.2)	277 (13.7)	65 (16.7)	
Drinking history, n (%)				< 0.001
No	2140 (89.2)	1813 (90.2)	327 (84.3)	
Yes	259 (10.8)	198 (9.8)	61 (15.7)	
Laboratory measurements				
HbA1c (%), Mean ± SD	8.4 ± 2.0	8.4 ± 2.0	8.3 ± 1.7	0.74
eGFR (mL/min/1.73 m²), Mean ± SD	112.8 ± 47.4	112.1 ± 46.9	116.6 ± 50.2	0.08
Triglycerides (mmol/L), Median (IQR)	1.4 (1.0, 2.2)	1.5 (1.0, 2.2)	1.4 (1.0, 2.1)	0.664
Total cholesterol (mmol/L), Mean ± SD	4.4 ± 1.5	4.4 ± 1.5	4.2 ± 1.3	0.071
HDL cholesterol (mmol/L), Mean ± SD	1.2 ± 0.4	1.2 ± 0.4	1.2 ± 0.3	0.159
LDL cholesterol (mmol/L), Median (IQR)	2.5 (1.8, 3.1)	2.5 (1.9, 3.1)	2.4 (1.8, 3.1)	0.114
Comorbidities				
Hypertension, n (%)				0.549
No	726 (29.6)	613 (29.8)	113 (28.3)	
Yes	1729 (70.4)	1443 (70.2)	286 (71.7)	
Cerebral infarction, n (%)				0.001
No	1394 (56.8)	1138 (55.3)	256 (64.2)	
Yes	1062 (43.2)	919 (44.7)	143 (35.8)	
Diabetic retinopathy, n (%)				< 0.001
No	2064 (84.0)	1753 (85.2)	311 (77.9)	
Yes	392 (16.0)	304 (14.8)	88 (22.1)	
Medications				
Metformin, n (%)				0.716
No	1485 (60.5)	1247 (60.6)	238 (59.6)	
Yes	971 (39.5)	810 (39.4)	161 (40.4)	
Sulfonylureas, n (%)				0.005
No	2178 (88.7)	1808 (87.9)	370 (92.7)	
Yes	278 (11.3)	249 (12.1)	29 (7.3)	
α-glucosidase inhibitors, n (%)				0.098
No	1841 (75.0)	1555 (75.6)	286 (71.7)	
Yes	615 (25.0)	502 (24.4)	113 (28.3)	
Thiazolidinediones, n (%)				0.049
No	2407 (98.0)	2021 (98.2)	386 (96.7)	
Yes	49 (2.0)	36 (1.8)	13 (3.3)	
GLP-1 analogues, n (%)				< 0.001
No	2414 (98.3)	2030 (98.7)	384 (96.2)	
Yes	42 (1.7)	27 (1.3)	15 (3.8)	
DPP-4 inhibitors, n (%)				0.087
No	2332 (95.0)	1960 (95.3)	372 (93.2)	
Yes	124 (5.0)	97 (4.7)	27 (6.8)	
Insulin.Use, n (%)				0.313
No	1583 (64.5)	1318 (64.1)	265 (66.8)	
Yes	870 (35.5)	738 (35.9)	132 (33.2)	

Data are presented as mean ± standard deviation, median (interquartile range), or number (percentage), as appropriate. Group comparisons were performed using the independent t-test, Mann–Whitney U test, or chi-square test, as appropriate. (p < 0.05) was considered statistically significant.

BMI, Body mass index; FHD, family history of diabetes; SGLT2i, sodium-glucose cotransporter 2 inhibitor; SD, standard deviation; IQR, interquartile range; HbA1c, glycated hemoglobin; eGFR, estimated glomerular filtration rate; HDL, high-density lipoprotein; LDL, low-density lipoprotein; GLP-1, glucagon-like peptide-1; DPP-4, dipeptidyl peptidase-4.

We evaluated sensitivity to unmeasured confounding by deriving E−values from the propensity score–adjusted RR (1.64; 95% CI 1.31–2.05); the point E−value (~2.66) represents the minimum strength of association an unmeasured confounder would need with both SGLT2i exposure and DPN to fully account for the observed effect.

Predefined subgroup analyses (sex; BMI <24 vs ≥24 kg/m2; age <60 vs ≥60; family history of diabetes; HbA1c <7% vs. ≥ 7%; insulin use) were performed with multivariable logistic models adjusted for **Table 1** covariates (excluding the stratifier). Interaction was tested by likelihood−ratio comparisons; adjusted ORs (95% CIs) are presented in a forest plot.

We performed a sensitivity analysis by removing diabetes duration from the propensity score model and repeating the matching and weighting procedures. Covariate balance was reassessed using standardized mean differences, and the association was re-estimated across all analytical models to evaluate the robustness of the primary findings.

Analysis was performed using R 4.2.2 (http://www.Rproject.org; The R Foundation, Vienna, Austria) and the Free Statistics software (version 2.3; Beijing FreeClinical Medical Technology Co., Ltd, Beijing, China).

## 3 Results

### 3.1 Baseline characteristics of the study population stratified by SGLT2 inhibitor use

Of the 2, 456 patients included, 399 (16.2%) received sodium–glucose cotransporter 2 inhibitors (SGLT2i). *Compared with non−users, those treated with SGLT2i were more often male, marginally younger, more likely to have drinking history, had fewer prior cerebral infarctions, and more frequently exhibited retinopathy (all P ≤ 0.004)* (**Table 1**). *The prevalence of IHD was 44.2% and DKD was 14.1%. SGLT2 inhibitor users had significantly higher prevalences of both IHD (52.9% vs. 42.5%, P < 0.001) and diabetic kidney disease (17.8% vs. 13.4%, P = 0.02)* (**Table 1**).

### 3.2 Association between SGLT2 inhibitor use and diabetic peripheral neuropathy in type 2 diabetes

The initial, unmatched population comprised 2, 456 patients (2, 057 without and 399 with SGLT2i). In the raw sample several baseline variables were imbalanced—female sex (SMD 0.322), age (0.16), history of alcohol use (0.188), diabetic retinopathy (0.189) and prior cerebral infarction (0.181)—pointing to potential confounding. After performing 1:1 propensity−score matching, 398 matched pairs were obtained and SMDs declined markedly (female sex 0.011; age 0.025; drinking history 0.028; retinopathy and cerebral infarction <0.001), with all covariates meeting the prespecified balance threshold (SMD < 0.1)(**Supplementary Table 1**; **Supplementary Figure 2**).

**Figure 2:**
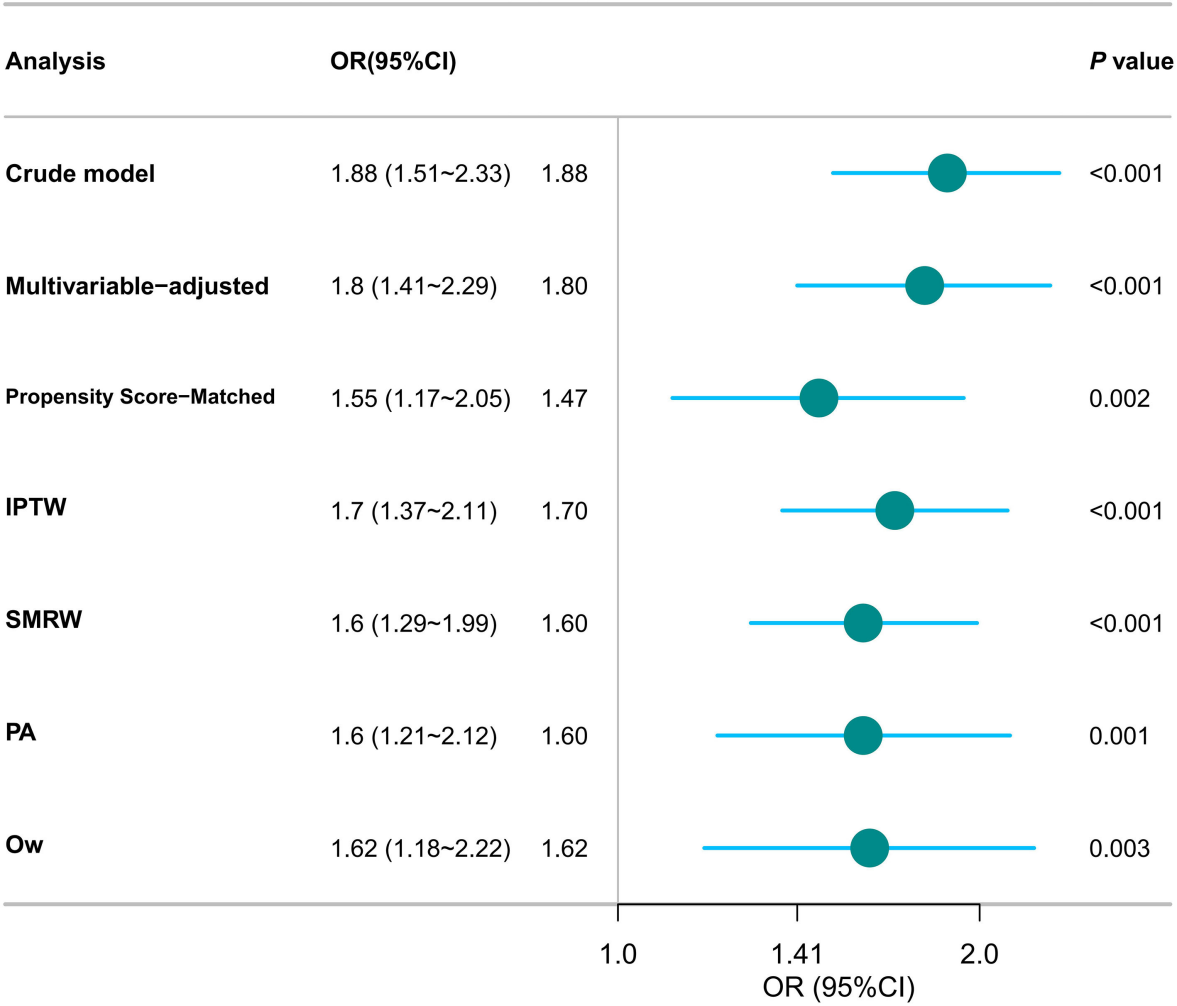
Association between SGLT2 inhibitor use and diabetic peripheral neuropathy across different analytic models. Forest plot displaying odds ratios (ORs) with 95% confidence intervals (CIs) for the association between SGLT2 inhibitor use and prevalent diabetic peripheral neuropathy (DPN) across multiple analytic approaches. Models include crude analysis, multivariable-adjusted analysis, propensity score adjustment (as a continuous covariate), 1:1 propensity score matching, and weighted methods (IPTW, SMRW, PA, and OW). The multivariable-adjusted model accounted for demographic characteristics, laboratory measurements, comorbidities, and concomitant glucose-lowering medications (covariates listed in **Table 1**). All propensity score-based models were constructed using the same set of covariates. The dashed vertical line represents the null effect (OR = 1.0). The point estimates and error bars indicate ORs and 95% CIs, respectively. Consistent positive associations were observed across all models, with SGLT2 inhibitor use showing significantly higher odds of DPN in each analytic approach. SGLT2i, sodium-glucose cotransporter 2 inhibitor; DPN, diabetic peripheral neuropathy; OR, odds ratio; CI, confidence interval; IPTW, inverse probability of treatment weighting; SMRW, standardized mortality ratio weighting; PA, Pairwise Algorithm; OW, overlap weighting.

**Figure 2** displays the relationship between SGLT2 inhibitor exposure and prevalent DPN across a range of analytic methods. Propensity score-based techniques produced odds ratios from 1.55 to 1.70 (all *P* < 0.01); one-to-one matching on the PS yielded the most conservative estimate (OR 1.55, 95% CI 1.17–2.05, *P* = 0.002), while weighting schemes (IPTW, SMRW, PS adjustment, OW) returned similar effect sizes (OR 1.60–1.70; all *P* ≤ 0.003). The unadjusted model indicated elevated odds of DPN among SGLT2i users (OR 1.88, 95% CI 1.51–2.33, *P* < 0.001), and multivariable adjustment only modestly reduced this association (OR 1.80, 95% CI 1.41–2.29, *P* < 0.001).

### 3.3 Sensitivity analysis

**Figure 3:**
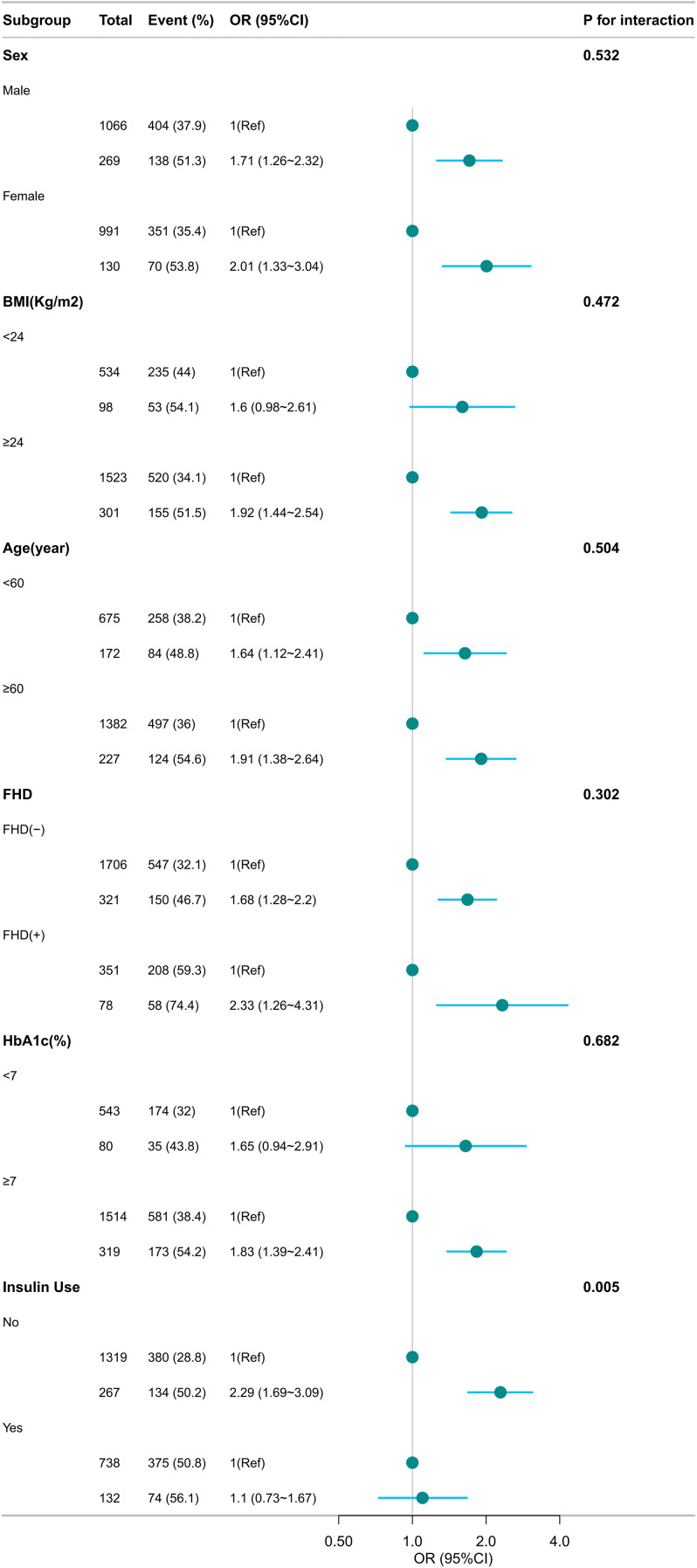
Forest plot of subgroup analyses for the association between SGLT2 inhibitor use and diabetic peripheral neuropathy. Forest plot displaying the odds ratios (ORs) and 95% confidence intervals (CIs) for the association between SGLT2 inhibitor use and diabetic peripheral neuropathy across predefined subgroups. Subgroup analyses were stratified by sex, body mass index (BMI; < 24 kg/m² vs. ≥ 24 kg/m²), age (< 60 years vs. ≥ 60 years), family history of diabetes (FHD), HbA1c(< 7% vs. ≥ 7%) and insulin use. ORs were estimated using logistic regression models adjusted for all covariates listed in **Table 1**, except for the stratification variable in each respective subgroup analysis. Horizontal lines represent 95% CIs. *P* values for interaction were calculated using likelihood ratio tests comparing models with and without interaction terms. A statistically significant interaction was observed only for insulin use (P for interaction = 0.005), suggesting that the association between SGLT2 inhibitors and DPN may vary depending on concomitant insulin therapy. All other subgroup analyses showed no significant interaction (P for interaction > 0.05 for all other variables), indicating that the association between SGLT2 inhibitor use and DPN was generally consistent across these subgroups. SGLT2i, sodium-glucose cotransporter 2 inhibitor; OR, odds ratio; CI, confidence interval; BMI, body mass index; FHD, family history of diabetes; DPN, diabetic peripheral neuropathy; HbA1c, glycated hemoglobin; Ref, reference category.

The E−value analysis produced a point estimate of 2.66 (lower limit 1.95), suggesting that only a confounder with a strong dual association (RR ≥ 2.66) with SGLT2i therapy and DPN could overturn the observed effect (**Supplementary Table 2**). We conducted subgroup analyses to determine whether the association between SGLT2 inhibitor use and DPN prevalence was consistent across predefined clinical strata (**Figure 3**). A statistically significant interaction was observed only for insulin use (P for interaction = 0.005), suggesting that the association between SGLT2 inhibitors and DPN may vary depending on concomitant insulin therapy. All other subgroup analyses showed no significant interaction (P for interaction > 0.05 for all other variables), indicating that the association between SGLT2 inhibitor use and DPN was generally consistent across these subgroups.

In the sensitivity analysis that excluded diabetes duration from all propensity score models and multivariable adjustment, the results remained materially unchanged across all analytical approaches. The association between SGLT2 inhibitor use and DPN persisted in the PSM analysis (OR, 1.41; 95% CI, 1.07–1.86; P = 0.016) and was consistent with the primary findings across all weighted models (IPTW: OR, 1.70; 95% CI, 1.37–2.11; SMRW: OR, 1.61; 95% CI, 1.30–1.99; PA: OR, 1.61; 95% CI, 1.21–2.13; OW: OR, 1.62; 95% CI, 1.19–2.22; all P < 0.05)(**Supplementary Tables 3**, **4**).

SGLT2 inhibitor use was significantly associated with higher odds of diabetic peripheral neuropathy in all models across five multiple imputed datasets, including unadjusted (OR, 1.88; 95% CI, 1.51–2.33), multivariable adjusted, and various propensity score–based analyses. The association remained robust after propensity score matching (OR, 1.42–1.77) and across weighted approaches, including IPTW (OR, 1.70–1.73), SMRW (OR, 1.59–1.62), PA (OR, 1.59–1.62), and OW (OR, 1.61–1.63), with all P values < 0.05 (**Supplementary Table 5**).

## 4 Discussion

In this cross-sectional study of 2, 456 patients with type 2 diabetes, SGLT2 inhibitor use was associated with significantly higher odds of prevalent diabetic peripheral neuropathy (DPN) after rigorous adjustment for confounding. Established risk factors for diabetic peripheral neuropathy include longer diabetes duration, poor glycemic control, older age, obesity, hypertension, dyslipidemia, and smoking. In our cross-sectional analysis, these variables were adjusted as confounders. In the propensity score-matched sample, SGLT2 inhibitor use was associated with higher odds of prevalent DPN compared with non-use (OR 1.47; 95% CI, 1.11–1.94; p = 0.007), corresponding to 47% higher odds in SGLT2i users. This positive association remained robust across a spectrum of analytic approaches, including multivariable adjustment, propensity score adjustment as a continuous covariate, and various weighting methods (IPTW, SMRW, OW), with ORs ranging from 1.60 to 1.80 (all *p* < 0.01). The combined results of subgroup analyses and E-value sensitivity testing indicated that the association was robust.

Metformin-associated vitamin B12 deficiency may worsen neuropathy, necessitating surveillance (20, 21). Rapid glycaemic correction with insulin carries a theoretical risk of treatment-induced neuropathy, yet confounding by indication remains problematic (22). Evidence for neuroprotection by GLP-1 agonists or thiazolidinediones is preclinical and inconclusive. Statin-neuropathy associations are inconsistent and confounded by vascular risk profiles (23).

Diabetes duration, defined by first documented diagnosis and patient self-report, is subject to recall bias and likely underestimates true disease duration, as suggested by the high baseline prevalence of complications. Reassuringly, a sensitivity analysis excluding diabetes duration from the propensity score model yielded results consistent with the primary analysis, supporting the robustness of our findings.

Our findings align with contemporary clinical data that have raised concerns regarding the potential neuropathic effects of SGLT2 inhibitors. Preclinical studies have demonstrated that SGLT2i can induce rapid glycemic improvement, which paradoxically may precipitate “treatment-induced neuropathy”—a phenomenon characterized by acute neuropathic pain following sudden glucose normalization (24, 25). In addition, the weight loss associated with SGLT2i therapy, while generally beneficial for cardiovascular risk reduction, may theoretically exacerbate pre-existing neuropathic symptoms in vulnerable patients (13). A *post-hoc* analysis of the CANVAS Program reported a numerically higher incidence of peripheral neuropathy-related adverse events in the canagliflozin group compared with placebo, though the difference did not reach statistical significance (26). Our study extends these observations by providing real-world evidence from a large, well-characterized population with T2DM, employing multiple propensity score-based methods to minimize confounding by indication—a major limitation of previous analyses.

However, our results appear to contrast with the prevailing hypothesis that SGLT2i may confer neuroprotective benefits through anti-inflammatory and antioxidative mechanisms. Several experimental studies have shown that SGLT2i can reduce oxidative stress in dorsal root ganglia neurons and improve nerve conduction velocity in diabetic animal models (12, 27). Moreover, secondary analyses of outcome trials have suggested potential benefits for other microvascular complications, such as diabetic kidney disease and retinopathy (28–31). The divergence between our findings and these mechanistic studies may be attributable to several factors. First, the neuroprotective effects observed in animal models may not translate directly to human clinical outcomes, particularly given the complex multifactorial pathophysiology of DPN. Second, the beneficial effects on oxidative stress and inflammation may be counterbalanced by other SGLT2i-induced metabolic shifts, such as rapid glucose lowering, ketogenesis, or electrolyte disturbances, which could adversely affect peripheral nerve function in susceptible individuals (24, 32). We lacked serial metabolic data (weight change, HbA1c trajectory, ketones, electrolytes) around SGLT2i initiation, precluding evaluation of whether treatment-induced metabolic shifts mediate our findings. Prospective studies with timed biomarker and nerve function assessments are needed to clarify these pathways.Third, the relatively short duration of follow-up in our cross-sectional design may capture early “on-treatment” effects rather than long-term protective effects, which may require extended exposure to manifest.

The biological plausibility of a detrimental association between SGLT2i and DPN is supported by several mechanistic pathways. DPN is a multifactorial condition involving oxidative stress, mitochondrial dysfunction, and microvascular ischemia (4, 33, 34). While SGLT2i are known to mitigate some of these pathways, the rapid correction of chronic hyperglycemia may induce a state of “metabolic stress” in neurons accustomed to hyperglycemic conditions, leading to paradoxical nerve injury (35). This phenomenon, termed “insulin neuritis” or treatment-induced neuropathy, has been well documented with rapid glycemic improvement achieved via intensive insulin therapy or bariatric surgery (36). SGLT2i, by inducing glucosuria and promoting substantial glucose lowering, may similarly precipitate this syndrome in a subset of patients. Additionally, SGLT2i-mediated volume depletion and electrolyte shifts could theoretically affect nerve conduction, particularly in patients with pre-existing autonomic neuropathy (37, 38).

This study has several important strengths. First, we employed a comprehensive propensity score-based analytical framework, including 1:1 matching and multiple weighting methods (IPTW, SMRW, OW), to rigorously control for a wide range of potential confounders, including demographic characteristics, laboratory measurements, comorbidities, and concomitant glucose-lowering medications. The balance achieved across groups, as evidenced by standardized mean differences below 0.1 after adjustment, substantially mitigates confounding by indication—a pervasive challenge in pharmacoepidemiologic studies. Second, the use of E-value analysis provides a quantitative assessment of the robustness of our findings against unmeasured confounding, enhancing the credibility of the observed association. Third, the inclusion of multiple sensitivity analyses, including subgroup evaluations and alternative propensity score techniques, consistently yielded similar effect estimates, supporting the internal validity of our results. Fourth, our study population was derived from a real-world clinical setting with standardized neurological assessments performed by trained personnel, increasing the clinical relevance and generalizability of the findings to routine practice.

Several limitations should be acknowledged. First, because the present study is cross−sectional and relied on routinely collected electronic medical record data, we were unable to implement a new−user longitudinal design and could not reliably determine duration or timing of SGLT2 inhibitor exposure relative to onset of diabetic peripheral neuropathy. Consequently, our results are subject to prevalent−user bias, potential survivor bias, and residual confounding (including confounding by indication). These limitations preclude causal inference regarding an effect of SGLT2 inhibitors on incident DPN. Although we employed rigorous confounding control, the possibility of reverse causation (i.e., patients with early DPN symptoms being preferentially prescribed SGLT2i) cannot be fully excluded. Prospective cohort studies or target trial emulations using new-user designs are warranted to confirm our findings. Second, despite comprehensive adjustment for measured confounders, residual confounding by unmeasured or imprecisely measured variables—such as the severity of glycemic control prior to SGLT2i initiation, duration of SGLT2i exposure, BMI, ketone bodies, and electrolyte measurements, Urinary Albumin-Creatinine Ratio, HOMA-IR or adherence to therapy—may persist. The E-value analysis suggests that only a strong unmeasured confounder could fully explain away the observed effect, but this does not definitively rule out residual confounding. Third, as dosing and duration data were incompletely recorded in this cross-sectional dataset, we were unable to assess dose–response relationships between SGLT2 inhibitor use and diabetic peripheral neuropathy. The absence of a dose-dependent effect in our analysis therefore does not preclude such a relationship. Fourth, DPN diagnosis in our study was based on standardized clinical assessment, which may have introduced misclassification if asymptomatic patients with subclinical neuropathy were misclassified as non-cases. However, the use of a rigorous diagnostic algorithm incorporating both symptoms and neurological signs likely minimized such misclassification. Fifth, the single-center design may limit generalizability to other populations or healthcare settings. Our findings should be interpreted in the context of the SGLT2 inhibitor landscape at our centre, where only dapagliflozin and empagliflozin were prescribed. Whether similar associations exist with other SGLT2 inhibitors—particularly those with distinct molecular structures or selectivity profiles—remains to be determined in multicentre studies with broader drug formularies. External validation in diverse, multicenter cohorts is needed. Sixth, as with any observational study, the associations identified do not establish causality; our findings should be interpreted as hypothesis-generating and require prospective designs or randomized trials with defined start dates and continuous follow-up to assess incidence and dose/duration relationships.

## 5 Conclusion

This cross−sectional study found that SGLT2 inhibitor use was associated with a higher prevalence of diabetic peripheral neuropathy among patients with type 2 diabetes. Due to the limitations inherent in the study design, however, residual confounding, reverse causation, and treatment−selection bias cannot be excluded and may partially or fully explain the observed association. These findings need further investigation into the potential mechanisms underlying this association, particularly the possible role of rapid glycemic improvement, metabolic stress, or treatment-induced neuropathy in susceptible individuals. While residual confounding cannot be fully excluded given the observational design, this study contributes to highlight the importance of balancing their established cardiovascular and renal benefits against potential neurological risks in the clinical management of type 2 diabetes.

## Data Availability

The original contributions presented in the study are included in the article/**Supplementary Material**. Further inquiries can be directed to the corresponding author.

## Supplementary material

Supplementary Figure 1 Standardized mean differences (SMDs) of covariates before and after propensity score-based adjustment. Standardized mean differences (SMDs) for all covariates across different analytic approaches: unmatched, propensity score matched (PSM), inverse probability of treatment weighting (IPTW), standardized mortality ratio weighting (SMRW), propensity score adjustment (PA), and overlap weighting (OW). The vertical dashed line indicates the conventional threshold of SMD < 0.1, representing adequate balance between the SGLT2 inhibitor and non-SGLT2 inhibitor groups. Covariates are ordered by SMD values in the unmatched analysis. Following propensity score-based adjustment, all covariates achieved SMDs below 0.1, indicating satisfactory balance across groups. SMD, standardized mean difference; IPTW, inverse probability of treatment weighting; SMRW, standardized mortality ratio weighting; PA, propensity score adjustment (continuous covariate adjustment); OW, overlap weighting; HbA1c, glycated hemoglobin; eGFR, estimated glomerular filtration rate; TG, triglycerides; HDL-C, high-density lipoprotein cholesterol; LDL-C, low-density lipoprotein cholesterol; GLP-1, glucagon-like peptide-1; DPP-4, dipeptidyl peptidase-4.

Supplementary Table 1 Baseline characteristics before and after propensity score matching. Data are presented as mean ± standard deviation (SD) or number (percentage), as appropriate. Standardized mean difference (SMD) was used to assess balance between groups. An SMD < 0.1 was considered indicative of adequate balance. Propensity score matching was performed using a 1:1 nearest neighbor matching algorithm with a caliper of 0.2 times the standard deviation of the propensity score. SGLT2i, sodium-glucose cotransporter 2 inhibitor; SMD, standardized mean difference; SD, standard deviation; HbA1c, glycated hemoglobin; eGFR, estimated glomerular filtration rate; HDL, high-density lipoprotein; LDL, low-density lipoprotein; GLP-1, glucagon-like peptide-1; DPP-4, dipeptidyl peptidase-4.

Supplementary Table 2 E-value analysis for unmeasured confounding. The E-value quantifies the minimum strength of association that an unmeasured confounder would need to have with both the exposure (SGLT2 inhibitor use) and the outcome (diabetic peripheral neuropathy), above and beyond the measured covariates, to fully explain away the observed association. The risk ratio (RR) was derived from the propensity score-adjusted model. An E-value of 2.47 for the point estimate indicates that an unmeasured confounder would need to be associated with both the exposure and outcome by a risk ratio of at least 2.47 (on average) to nullify the observed effect. The E-value for the lower bound of the 95% confidence interval (1.95) provides a sensitivity assessment for the most conservative estimate. CI, confidence interval; RR, risk ratio.

Supplementary Table 3 Association between SGLT2 inhibitor use and DPN across different analytical models. The reference group consists of patients not receiving SGLT2 inhibitors. The multivariable-adjusted model and propensity score (PS) models were adjusted for all baseline covariates listed in **Supplementary Table 1**, including: sex, age, body mass index (BMI), family history of diabetes, smoking history, history of drinking, HbA1c, estimated glomerular filtration rate (eGFR), triglycerides, total cholesterol, high-density lipoprotein cholesterol (HDL-C), low-density lipoprotein cholesterol (LDL-C), hypertension, cerebral infarction, diabetic retinopathy, and the use of biguanides, sulfonylureas, α-glucosidase inhibitors, thiazolidinediones, GLP-1 analogues, DPP-4 inhibitors, and insulin. The PS-adjusted model included the propensity score as a covariate. Propensity score-matched analysis was performed using a 1:1 nearest-neighbor matching algorithm without replacement. OR, odds ratio; CI, confidence interval; IPTW, inverse probability of treatment weighting; SMRW, standardized mortality ratio weighting; PA, propensity score stratification/overlap weighting; OW, overlap weighting; SGLT2, sodium-glucose cotransporter-2.

Supplementary Table 4 Baseline characteristics of the study sample stratified by SGLT2 inhibitor use before and after propensity score matching. Data are presented as mean ± standard deviation (SD) or number (percentage), as appropriate. Standardized mean difference (SMD) was used to assess balance between groups. An SMD < 0.1 was considered indicative of adequate balance. Propensity score matching was performed using a 1:1 nearest neighbor matching algorithm with a caliper of 0.2 times the standard deviation of the propensity score. SGLT2i, sodium-glucose cotransporter 2 inhibitor; SMD, standardized mean difference; SD, standard deviation; HbA1c, glycated hemoglobin; eGFR, estimated glomerular filtration rate; HDL, high-density lipoprotein; LDL, low-density lipoprotein; GLP-1, glucagon-like peptide-1; DPP-4, dipeptidyl peptidase-4.

Supplementary Table 5 Association between SGLT2 inhibitor use and diabetic peripheral neuropathy in type 2 diabetes patients assessed by different propensity score methods across five imputed datasets. OR, odds ratio; CI, confidence interval; PS, propensity score; IPTW, inverse probability of treatment weighting; SMRW, standardized mortality ratio weighting; PA, propensity score matching with replacement using the propensity score algorithm; OW, overlap weighting; SGLT2, sodium-glucose cotransporter 2; T2DM, type 2 diabetes mellitus. Data 1–5 represent the results derived from five multiple imputed datasets. The crude model was unadjusted for any covariates. The multivariable adjusted model accounted for baseline demographic and clinical covariates(same as the main result). PS adjusted, PS matched, IPTW, SMRW, PA, and OW models were adjusted for the propensity score derived from the same set of covariates. All P values are two-sided with statistical significance set at P < 0.05.
