# Supplementary material for: The association between SGLT2 inhibitor use and diabetic peripheral neuropathy in type 2 diabetes: a cross-sectional study
Source: Front Endocrinol (Lausanne). 2026 Jul 24;17:1860467. doi: 10.3389/fendo.2026.1860467 (PMC13447137; doi:10.3389/fendo.2026.1860467)
Supplement: Supplementary file 1 [file Image1.pdf]

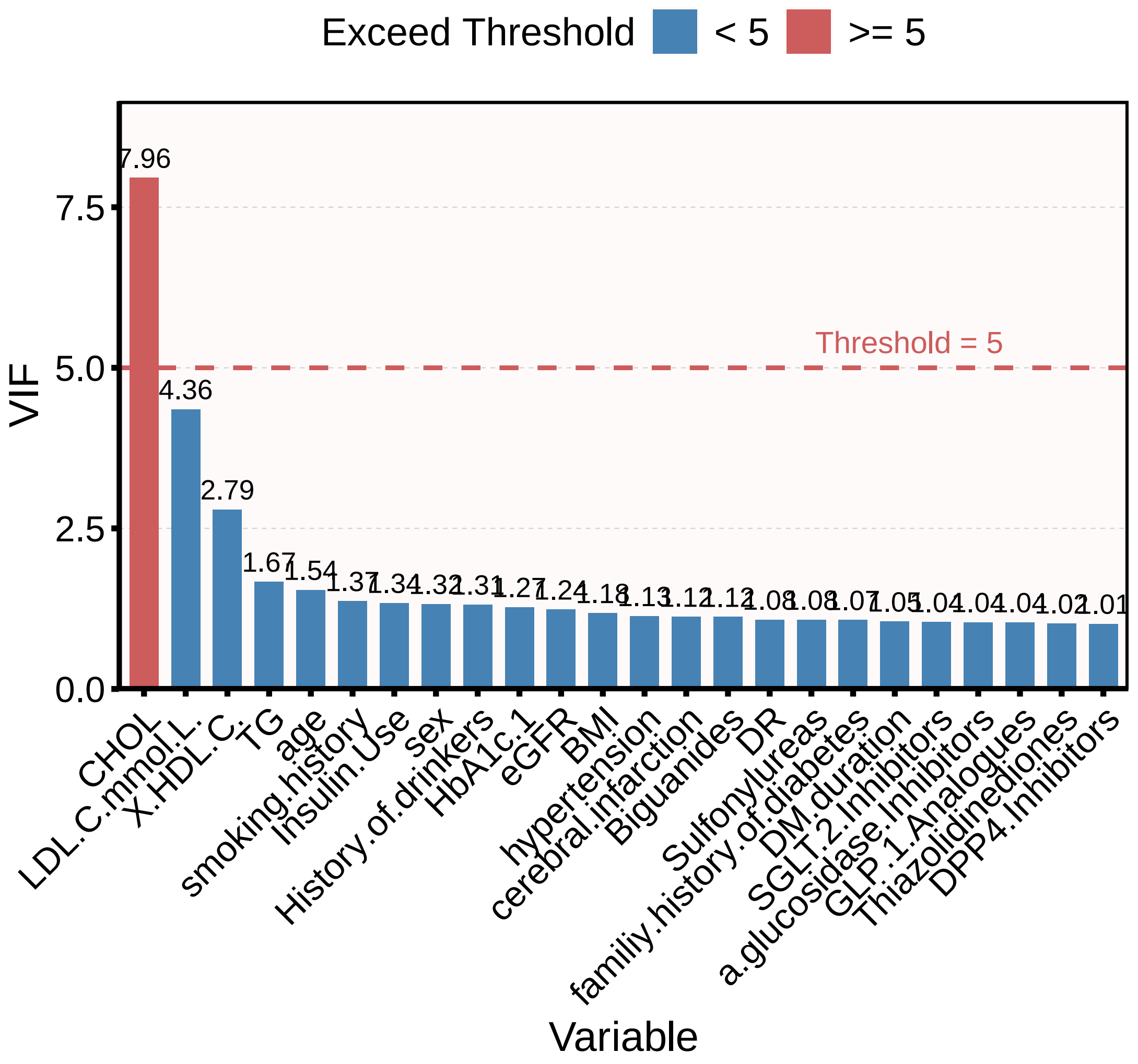

Exceed Threshold
< 5
>= 5
VIF
Threshold = 5
7.96
4.36
2.79
1.67
1.54
1.37
1.34
1.32
1.31
1.27
1.24
1.18
1.13
1.12
1.12
1.08
1.08
1.07
1.05
1.04
1.04
1.04
1.02
1.01
0.0
2.5
5.0
7.5
CHOL
LDL.C.mmol.L.
X.HDL.C.
TG
age
smoking.history
Insulin.Use
sex
History.of.drinkers
HbA1c.1
eGFR
BMI
hypertension
cerebral.infarction
Biguanides
DR
Sulfonylureas
familiy.history.of.diabetes
DM.duration
SGLT.2.Inhibitors
a.glucosidase.Inhibitors
GLP.1.Analogues
Thiazolidinediones
DPP4.Inhibitors
Variable
