# Supplementary material for: The association between SGLT2 inhibitor use and diabetic peripheral neuropathy in type 2 diabetes: a cross-sectional study
Source: Front Endocrinol (Lausanne). 2026 Jul 24;17:1860467. doi: 10.3389/fendo.2026.1860467 (PMC13447137; doi:10.3389/fendo.2026.1860467)
Supplement: Supplementary file 2 [file Image2.pdf]

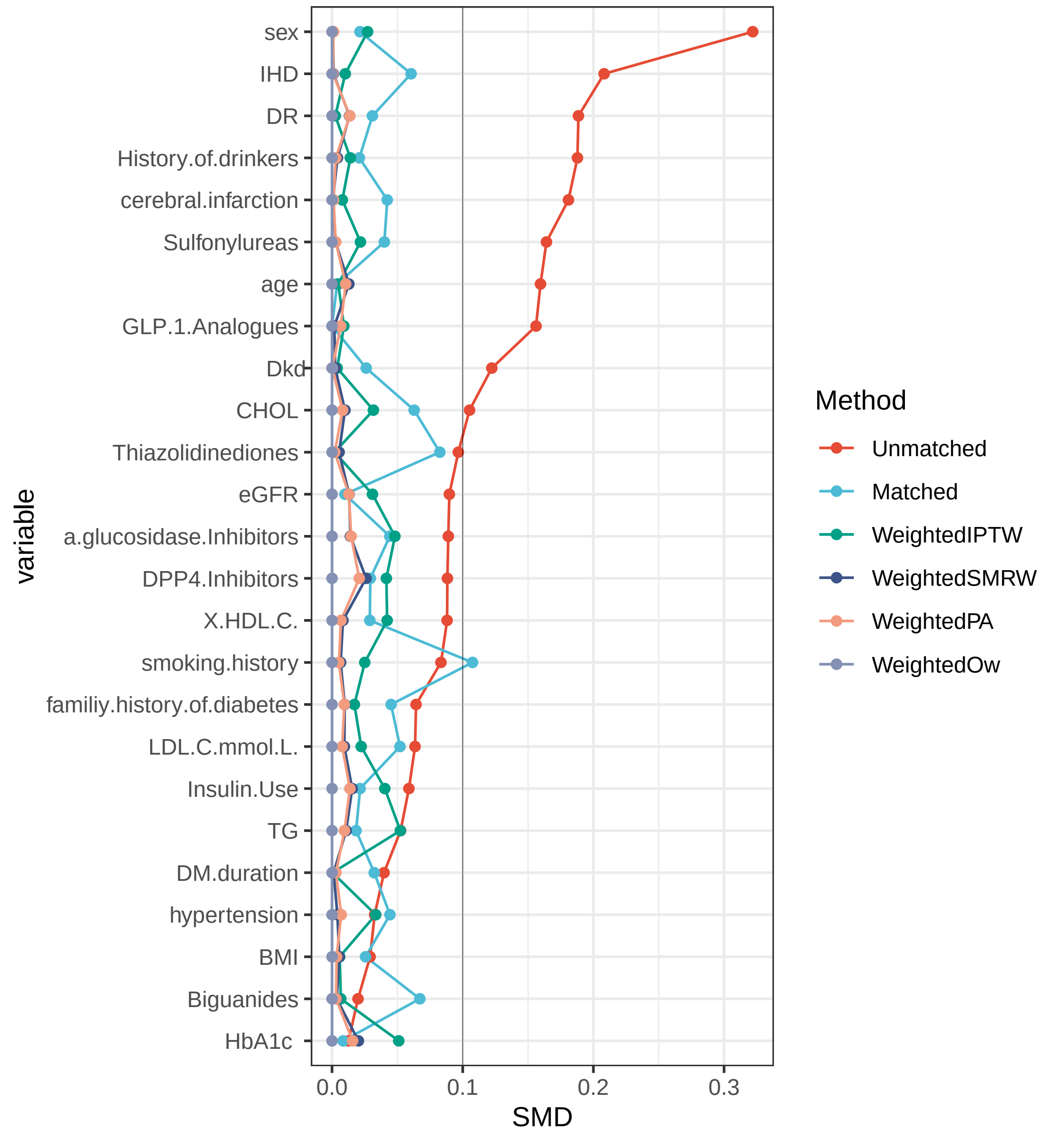

sex
IHD
DR
History.of.drinkers
cerebral.infarction
Sulfonylureas
age
GLP.1.Analogues
Dkd
CHOL
Thiazolidinediones
eGFR
a.glucosidase.Inhibitors
DPP4.Inhibitors
X.HDL.C.
smoking.history
familiy.history.of.diabetes
LDL.C.mmol.L.
Insulin.Use
TG
DM.duration
hypertension
BMI
Biguanides
HbA1c
variable
0.0
0.1
0.2
0.3
SMD
Method
Unmatched
Matched
WeightedIPTW
WeightedSMRW
WeightedPA
WeightedOw
