# Supplementary material for: The association between SGLT2 inhibitor use and diabetic peripheral neuropathy in type 2 diabetes: a cross-sectional study
Source: Front Endocrinol (Lausanne). 2026 Jul 24;17:1860467. doi: 10.3389/fendo.2026.1860467 (PMC13447137; doi:10.3389/fendo.2026.1860467)
Supplement: Supplementary file 3 [file Table1.doc]

**Supplementary Table 1. Baseline characteristics before and after propensity score matching**

| Variable | Before matching | | SMD | After matching | | SMD |
| --- | --- | --- | --- | --- | --- | --- |
|  | Non-SGLT2i group  (n = 2057) | SGLT2i group  (n = 399) |  | Non-SGLT2i group  (n = 398) | SGLT2i group  (n = 398) |  |
| Demographic characteristics |  |  |  |  |  |  |
| Female sex, n (%) | 991 (48.2) | 130 (32.6) | 0.322 | 128 (32.2) | 130 (32.7) | 0.016 |
| Age (years), Mean ± SD | 63.13 ± 11.61 | 61.34 ± 10.90 | 0.16 | 61.63 ± 12.13 | 61.33 ± 10.92 | 0.036 |
| Body mass index (kg/m²), Mean ± SD | 26.34 ± 5.93 | 26.20 ± 3.50 | 0.029 | 26.16 ± 3.53 | 26.19 ± 3.50 | 0.055 |
| Diabetes duration (years), Mean ± SD | 4.73 ± 5.36 | 4.95 ± 5.98 | 0.04 | 4.72 ± 5.27 | 4.92 ± 5.96 | 0.023 |
| Family history of diabetes, n (%) | 351 (17.1) | 78 (19.5) | 0.064 | 79 (19.8) | 78 (19.6) | 0.08 |
| Smoking history, n (%) | 279 (13.6) | 66 (16.5) | 0.083 | 57 (14.3) | 66 (16.6) | 0.007 |
| Drinking history, n (%) | 201 (9.8) | 64 (16.0) | 0.188 | 59 (14.8) | 63 (15.8) | 0.02 |
| Laboratory measurements |  |  |  |  |  |  |
| HbA1c (%), Mean ± SD | 8.34 ± 1.92 | 8.32 ± 1.65 | 0.013 | 8.44 ± 1.98 | 8.32 ± 1.65 | 0.05 |
| eGFR (mL/min/1.73 m²), Mean ± SD | 112.11 ± 46.61 | 116.46 ± 50.24 | 0.09 | 115.31 ± 44.29 | 116.50 ± 50.29 | 0.024 |
| Triglycerides (mmol/L), Mean ± SD | 2.01 ± 2.34 | 1.90 ± 1.85 | 0.052 | 1.84 ± 1.68 | 1.90 ± 1.85 | 0.02 |
| HDL cholesterol (mmol/L), Mean ± SD | 1.21 ± 0.45 | 1.18 ± 0.33 | 0.088 | 1.18 ± 0.30 | 1.18 ± 0.33 | 0.007 |
| LDL cholesterol (mmol/L), Mean ± SD | 2.66 ± 3.63 | 2.49 ± 0.97 | 0.064 | 2.55 ± 0.95 | 2.49 ± 0.98 | 0.041 |
| Comorbidities |  |  |  |  |  |  |
| Hypertension, n (%) | 1444 (70.2) | 286 (71.7) | 0.033 | 292 (73.4) | 286 (71.9) | 0.05 |
| Cerebral infarction, n (%) | 919 (44.7) | 143 (35.8) | 0.181 | 143 (35.9) | 143 (35.9) | 0.063 |
| Diabetic retinopathy, n (%) | 304 (14.8) | 88 (22.1) | 0.189 | 87 (21.9) | 87 (21.9) | 0.036 |
| Medications |  |  |  |  |  |  |
| Metformin, n (%) | 810 (39.4) | 161 (40.4) | 0.02 | 150 (37.7) | 161 (40.5) | 0.062 |
| Sulfonylureas, n (%) | 249 (12.1) | 29 (7.3) | 0.164 | 25 (6.3) | 29 (7.3) | 0.01 |
| α-glucosidase inhibitors, n (%) | 502 (24.4) | 113 (28.3) | 0.089 | 127 (31.9) | 113 (28.4) | <0.001 |
| Thiazolidinediones, n (%) | 36 (1.8) | 13 (3.3) | 0.097 | 13 (3.3) | 13 (3.3) | 0.045 |
| GLP-1 analogues, n (%) | 27 (1.3) | 15 (3.8) | 0.156 | 11 (2.8) | 14 (3.5) | 0.059 |
| DPP-4 inhibitors, n (%) | 97 (4.7) | 27 (6.8) | 0.088 | 25 (6.3) | 27 (6.8) | 0.01 |
| Insulin use, n (%) | 738 (35.9) | 132 (33.1) | 0.059 | 138 (34.7) | 132 (33.2) | 0.063 |

Notes:Data are presented as mean ± standard deviation (SD) or number (percentage), as appropriate. Standardized mean difference (SMD) was used to assess balance between groups. An SMD < 0.1 was considered indicative of adequate balance. Propensity score matching was performed using a 1:1 nearest neighbor matching algorithm with a caliper of 0.2 times the standard deviation of the propensity score.

Abbreviations: SGLT2i, sodium-glucose cotransporter 2 inhibitor; SMD, standardized mean difference; SD, standard deviation; HbA1c, glycated hemoglobin; eGFR, estimated glomerular filtration rate; HDL, high-density lipoprotein; LDL, low-density lipoprotein; GLP-1, glucagon-like peptide-1; DPP-4, dipeptidyl peptidase-4.
