# Supplementary material for: The association between SGLT2 inhibitor use and diabetic peripheral neuropathy in type 2 diabetes: a cross-sectional study
Source: Front Endocrinol (Lausanne). 2026 Jul 24;17:1860467. doi: 10.3389/fendo.2026.1860467 (PMC13447137; doi:10.3389/fendo.2026.1860467)
Supplement: Supplementary file 4 [file Table2.doc]

**Supplementary Table 2. E-value analysis for unmeasured confounding**

| Indicator (Item) | Value (Point) | Lower Limit of 95% Confidence Interval (Lower) | Upper Limit of 95% Confidence Interval (Upper) |
| --- | --- | --- | --- |
| Relative Risk (RR) | 1.55 | 1.17 | 2.05 |
| Effect Size Value (E-values) | 2.47 | 1.62 | NA |

**Notes:** The E-value quantifies the minimum strength of association that an unmeasured confounder would need to have with both the exposure (SGLT2 inhibitor use) and the outcome (diabetic peripheral neuropathy), above and beyond the measured covariates, to fully explain away the observed association. The risk ratio (RR) was derived from the propensity score-adjusted model. An E-value of 2.47 for the point estimate indicates that an unmeasured confounder would need to be associated with both the exposure and outcome by a risk ratio of at least 2.47 (on average) to nullify the observed effect. The E-value for the lower bound of the 95% confidence interval (1.95) provides a sensitivity assessment for the most conservative estimate.

**Abbreviations:** CI, confidence interval; RR, risk ratio.
