# Supplementary material for: The association between SGLT2 inhibitor use and diabetic peripheral neuropathy in type 2 diabetes: a cross-sectional study
Source: Front Endocrinol (Lausanne). 2026 Jul 24;17:1860467. doi: 10.3389/fendo.2026.1860467 (PMC13447137; doi:10.3389/fendo.2026.1860467)
Supplement: Supplementary file 5 [file Table3.doc]

**Supplementary Table 3. Association Between SGLT2 Inhibitor Use and DPN Across Different Analytical Models**

| Models | OR (95% CI) | P value |
| --- | --- | --- |
| Unmatched, crude | 1.88 (1.51–2.33) | <0.001 |
| Multivariable-adjusted | 1.80 (1.42–2.30) | <0.001 |
| Propensity score-adjusted | 1.64 (1.31–2.05) | <0.001 |
| Propensity score-matched | 1.41 (1.07–1.86) | 0.016 |
| IPTW | 1.70 (1.37–2.11) | <0.001 |
| SMRW | 1.61 (1.30–1.99) | <0.001 |
| PA | 1.61 (1.21–2.13) | 0.001 |
| OW | 1.62 (1.19–2.22) | 0.002 |

**Notes:** The reference group consists of patients not receiving SGLT2 inhibitors. The multivariable-adjusted model and propensity score (PS) models were adjusted for all baseline covariates listed in Table 2, including: sex, age, body mass index (BMI), family history of diabetes, smoking history, history of drinking, HbA1c, estimated glomerular filtration rate (eGFR), triglycerides, total cholesterol, high-density lipoprotein cholesterol (HDL-C), low-density lipoprotein cholesterol (LDL-C), hypertension, cerebral infarction, diabetic retinopathy, and the use of biguanides, sulfonylureas, α-glucosidase inhibitors, thiazolidinediones, GLP-1 analogues, DPP-4 inhibitors, and insulin. The PS-adjusted model included the propensity score as a covariate. Propensity score-matched analysis was performed using a 1:1 nearest-neighbor matching algorithm without replacement.

**Abbreviations:** OR, odds ratio; CI, confidence interval; IPTW, inverse probability of treatment weighting; SMRW, standardized mortality ratio weighting; PA, propensity score stratification/overlap weighting; OW, overlap weighting; SGLT2, sodium-glucose cotransporter-2
