# Supplementary material for: The association between SGLT2 inhibitor use and diabetic peripheral neuropathy in type 2 diabetes: a cross-sectional study
Source: Front Endocrinol (Lausanne). 2026 Jul 24;17:1860467. doi: 10.3389/fendo.2026.1860467 (PMC13447137; doi:10.3389/fendo.2026.1860467)
Supplement: Supplementary file 7 [file Table5.doc]

**Supplementary Table 5. Association Between SGLT2 Inhibitor Use and Diabetic Peripheral Neuropathy in Type 2 Diabetes Patients Assessed by Different Propensity Score Methods Across Five Imputed Datasets**

| Models | Data 1 | | Data 2 | | Data 3 | | Data 4 | | Data 5 | |
| --- | --- | --- | --- | --- | --- | --- | --- | --- | --- | --- |
|  | OR (95%CI) | *P* value | OR (95%CI) | *P* value | OR (95%CI) | *P* value | OR (95%CI) | *P* value | OR (95%CI) | *P* value |
| Unmatched.crude | 1.88 (1.51~2.33) | <0.001 | 1.88 (1.51~2.33) | <0.001 | 1.88 (1.51~2.33) | <0.001 | 1.88 (1.51~2.33) | <0.001 | 1.88 (1.51~2.33) | <0.001 |
| Multivariable.adjusted | 1.81 (1.42~2.31) | <0.001 | 1.78 (1.4~2.27) | <0.001 | 1.81 (1.42~2.31) | <0.001 | 1.79 (1.41~2.28) | <0.001 | 1.79 (1.4~2.28) | <0.001 |
| PropensityScore.adjusted | 1.65 (1.32~2.05) | <0.001 | 1.63 (1.3~2.03) | <0.001 | 1.64 (1.32~2.05) | <0.001 | 1.63 (1.31~2.04) | <0.001 | 1.63 (1.31~2.04) | <0.001 |
| PropensityScore.Matched | 1.42 (1.08~1.88) | 0.013 | 1.58 (1.19~2.09) | 0.001 | 1.77 (1.34~2.35) | <0.001 | 1.75 (1.32~2.32) | <0.001 | 1.44 (1.09~1.9) | 0.011 |
| Weighted.IPTW | 1.7 (1.37~2.11) | <0.001 | 1.71 (1.38~2.12) | <0.001 | 1.71 (1.38~2.12) | <0.001 | 1.73 (1.39~2.14) | <0.001 | 1.71 (1.37~2.12) | <0.001 |
| Weighted.SMRW | 1.62 (1.31~2.01) | <0.001 | 1.59 (1.29~1.98) | <0.001 | 1.62 (1.3~2) | <0.001 | 1.6 (1.29~1.99) | <0.001 | 1.6 (1.29~1.99) | <0.001 |
| Weighted.PA | 1.62 (1.22~2.14) | 0.001 | 1.59 (1.2~2.11) | 0.001 | 1.61 (1.22~2.13) | 0.001 | 1.6 (1.21~2.12) | 0.001 | 1.6 (1.21~2.12) | 0.001 |
| Weighted.Ow | 1.63 (1.19~2.23) | 0.002 | 1.61 (1.18~2.21) | 0.003 | 1.63 (1.19~2.23) | 0.002 | 1.62 (1.18~2.21) | 0.003 | 1.62 (1.18~2.21) | 0.003 |

**Abbreviations:** OR, odds ratio; CI, confidence interval; PS, propensity score; IPTW, inverse probability of treatment weighting; SMRW, standardized mortality ratio weighting; PA, propensity score matching with replacement using the propensity score algorithm; OW, overlap weighting; SGLT2, sodium-glucose cotransporter 2; T2DM, type 2 diabetes mellitus.

**Notes:** Data 1–5 represent the results derived from five multiple imputed datasets. The crude model was unadjusted for any covariates. The multivariable adjusted model accounted for baseline demographic and clinical covariates(same as the main result). PS adjusted, PS matched, IPTW, SMRW, PA, and OW models were adjusted for the propensity score derived from the same set of covariates. All P values are two-sided with statistical significance set at P < 0.05.
